# Transjugular intrahepatic collateral-systemic shunt is effective for cavernous transformation of the portal vein with variceal bleeding

**DOI:** 10.1007/s12072-023-10522-z

**Published:** 2023-04-25

**Authors:** Jun Tie, Xiaoyuan Gou, Chuangye He, Kai Li, Xulong Yuan, Wenyuan Jia, Jing Niu, Na Han, Jiao Xu, Ying Zhu, Wenlan Wang

**Affiliations:** 1 National Clinical Research Center for Digestive Diseases and Xijing Hospital of Digestive Diseases, Xijing Hospital, Air Force Medical University, Xi’an, 710032 Shaanxi China; 2 Department of Aerospace Hygiene, School of Aerospace Medicine, Air Force Medical University, Xi’an, 710032 Shaanxi China

**Keywords:** Occlusive portal vein thrombosis, Cavernous transformation, Refractory variceal bleeding, Transjugular intrahepatic portosystemic shunt

## Abstract

**Background:**

The transjugular intrahepatic portal collateral-systemic shunt (transcollateral TIPS) is used to treat portal hypertension-related complications in patients with cavernous transformation of the portal vein (CTPV) and whose main portal vein cannot be recanalized. It is still not clear whether transcollateral TIPS can be as effective as portal vein recanalization–transjugular intrahepatic portosystemic shunt (PVR–TIPS). This study aimed to evaluate the efficacy and safety of transcollateral TIPS in the treatment of refractory variceal bleeding with CTPV.

**Methods:**

Patients with refractory variceal bleeding caused by CTPV were selected from the database of consecutive patients treated with TIPS in Xijing Hospital from January 2015 to March 2022. They were divided into the transcollateral TIPS group and the PVR–TIPS group. The rebleeding rate, overall survival, shunt dysfunction, overt hepatic encephalopathy (OHE) and operation-related complications were analyzed.

**Results:**

A total of 192 patients were enrolled, including 21 patients with transcollateral TIPS and 171 patients with PVR–TIPS. Compared with the patients with PVR–TIPS, the patients with transcollateral TIPS had more noncirrhosis (52.4 vs. 19.9%, *p* = 0.002), underwent fewer splenectomies (14.3 vs. 40.9%, *p* = 0.018), and had more extensive thromboses (38.1 vs. 15.2%, *p* = 0.026). There were no differences in rebleeding, survival, shunt dysfunction, or operation-related complication rates between the transcollateral TIPS and PVR–TIPS groups. However, the OHE rate was significantly lower in the transcollateral TIPS group (9.5 vs. 35.1%, *p* = 0.018).

**Conclusion:**

Transcollateral TIPS is an effective treatment for CTPV with refractory variceal bleeding.

**Supplementary Information:**

The online version contains supplementary material available at 10.1007/s12072-023-10522-z.

## Introduction

Cavernous transformation of the portal vein (CTPV) is the most serious status of portal thrombosis, and is often accompanied by a series of complications of portal hypertension, such as hematemesis, hematochezia, ascites, and intestinal ischemia [1]. Both the thrombus itself and the complications caused by thrombus, especially portal hypertension variceal bleeding, do not efficiently respond to drug and endoscopic treatment [2, 3]. Transjugular intrahepatic portosystemic shunt (TIPS) can reduce the thrombosis load, restore portal vein blood flow, decrease portal pressure, enhance patient eligibility for liver transplantation, and improve the prognosis of patients. Therefore, TIPS has become an effective method for complete portal thrombosis with CTPV refractory to medical and endoscopic therapy [3–7] and is recommended in several guidelines [8–11].

TIPS for CTPV is more technically difficult than conventional TIPS. During TIPS for CTPV, the portosystemic shunt can only be established on the basis of opening the blocked main portal vein (MPV). Whether the blocked MPV can be recanalized is the decisive factor for the success or failure of TIPS. In previous studies, the success rate of TIPS varied greatly both cirrhotic [12, 13] and noncirrhotic [3, 14] CTPV. This may be due to the heterogeneity of portal vein conditions among these studies, as well as the heterogeneity of the operators’ skills. In fact, even for experienced experts, the success rate of TIPS in patients with CTPV is unlikely to be 100%. In some patients with CTPV, TIPS failed because the MPV could not be recanalized. In these cases, using large, cavernous collateral vessels to establish portosystemic shunts may be an alternative option [15–17], which is called transcollateral TIPS. However, the previous studies on transcollateral TIPS were all case reports. The efficacy of transcollateral TIPS for portal hypertension-related complications has not been uniform. Whether transcollateral TIPS is as effective as portal vein recanalization–transjugular intrahepatic portosystemic shunt (PVR–TIPS) in patients with CTPV remains unclear. Thus, this study aimed to evaluate the efficacy and safety of transcollateral TIPS on portal hypertension complications in patients with CTPV.

## Methods

### Study design

This was a retrospective cohort study. The study was approved by the Medical Ethics Committee of the First Affiliated Hospital of the Air Force Medical University. The enrolled patients were consecutive patients with CTPV complicated by refractory variceal bleeding who were treated with TIPS in Xijing Hospital (First Affiliated Hospital of the Air Force Medical University) from January 2015 to March 2022. The last patient enrolled was followed for more than 6 months. The inclusion criteria were as follows: (1) chronic obstructive main portal vein thrombosis (PVT) with cavernous formation proven by at least one imaging examination (B-ultrasound, computed tomography, magnetic resonance imaging); (2) patients with esophageal and/or gastric variceal bleeding refractory to drug and endoscopic treatment; and (3) patients successfully treated with TIPS. The exclusion criteria were as follows: (1) malignancy (including hepatocellular carcinoma) or other shortened lifespan diseases; (2) common contraindications of TIPS, such as heart failure NYHA grade ≥ III, spontaneous bacterial peritonitis, human immunodeficiency virus infection or acquired immune deficiency syndrome-related diseases; and (3) no follow-up data.

The patients eligible for inclusion were selected and divided into a transcollateral TIPS group and a PVR–TIPS group according to the TIPS style. Then, the transcollateral TIPS group was used as the experimental group, and the PVR–TIPS group was the control group. The preoperative and postoperative PPG, rebleeding rate, shunt dysfunction, encephalopathy, overall survival and operation-related complications were analyzed between the patients with transcollateral TIPS and PVR–TIPS.

### Endpoints and definitions

The primary endpoint was variceal rebleeding, which was defined based on the recommendations in the Baveno V consensus [18]. The secondary endpoints included preoperative and postoperative PPG, shunt dysfunction, encephalopathy, overall survival and operation-related complications. Overt hepatic encephalopathy (OHE) was diagnosed according to the current guidelines.

CTPV was defined as gross porto-portal collaterals without the original MPV seen [19]. Liver cirrhosis and noncirrhosis were diagnosed as documented by a previous liver biopsy or a combination of usual clinical signs and biochemical parameters [20]. The extent of thrombosis was diagnosed by both computerized tomography (CT) and B-ultrasound examinations. The collaterals were confirmed by CT and angiography. Liver function damage was defined as a Child‒Pugh score increase of more than 1 point.

TIPS eligibility in patients with CTPV was as follows: (1) complete portal vein thrombosis with patent splenic vein and/or mesenteric vein; (2) complete portal and mesenteric vein thrombosis with distal patency of the splenic vein; and (3) complete portal vein thrombosis and splenectomy with distal patency of the mesenteric vein. TIPS ineligibility in patients with CTPV was CTPV and diffuse thrombosis of the splenic vein and mesenteric vein (Supplementary Fig. 1).

### TIPS procedure details and technique

All patients were given drug analgesia. The analgesia method was as follows: the patient was injected with 50 mg Pethidini hydrochloridum at the beginning of the TIPS, and lidocaine hydrochloride injection was used for local anesthesia at the skin piercing site. The types of TIPS that were performed were divided into two kinds. One was the PVR–TIPS technique. This method recanalizes the occluded MPV through transjugular, transhepatic, and/or transsplenic access and then finishes the TIPS. The other was the transcollateral TIPS technique. For the patients whose MPV could not be recanalized and who had enlarged collateral vessels, the collateral vessels were used to establish the portosystemic shunt. For both PVR–TIPS and transcollateral TIPS, we used 8 mm diameter stents. For PVR–TIPS, we first planted an 8 × 80 mm VIATORR stent (Gore, Arizona, USA) in the distal end, followed by an 8 mm diameter, 80–100 mm length Fluency stent (BARD, New Jersey, USA). In almost all patients, the longest VIATORR stent was also insufficient to cover the thrombus and hepatic parenchyma, and a second stent was needed. For patients with complete portal vein thrombosis accompanied by splenic vein thrombosis, we first planted the stents from the initiation of the portal vein as described above, and then recanalized the splenic vein using a balloon and/or bare stent (Cook, Chicago, USA). The length of the bare stent was determined by the extent and grade of the splenic vein thrombosis. For transcollateral TIPS, the treatment was similar to that of PVR–TIPS. The diameter of the collateral vessels was greater than or equal to 6 mm, and there were more than 2 cm straight vessels. All patients were initially treated with PVR–TIPS. Transcollateral TIPS was performed only when the portal vein could not be opened (Supplementary Fig. 2).

Anticoagulant therapy was used in cirrhotic patients with residual thrombosis after TIPS, as well as in all noncirrhotic patients. Low-molecular-weight heparin or warfarin was applied for anticoagulation.

### Measurements of PPG

The patients in this study had complete PVT, so the PPG of these patients was measured differently from that of the patients without portal thrombosis. The key point of PPG measurement in patients with portal thrombosis lies in the location of portal pressure measurement, which vary depending on the extent of the thrombus and the type of TIPS. For patients with transcollateral TIPS and the collateral vessel directly communicating with variceal veins, the portal pressure measurement site was located 2–5 cm distal to the collateral vessel puncture site (Supplementary Fig. 3A). For other patients, the portal pressure measurement site was located at an open blood vessel 2–5 cm distal to the thrombus. For patients with complete portal vein thrombosis with patent splenic vein and mesenteric vein, the portal pressure measurement site was located at the superior mesenteric vein (Supplementary Fig. 3B) or splenic vein (Supplementary Fig. 3C) 2–5 cm from the thrombosis; for patients with complete portal and mesenteric vein thrombosis with distal patency of the splenic vein, the portal pressure measurement site was located at the splenic vein (Supplementary Fig. 3D) 2–5 cm from the thrombosis; and for patients with complete portal vein thrombosis and splenectomy with distal patency of the mesenteric vein, the portal pressure measurement site was located at the superior mesenteric vein (Supplementary Fig. 3E) 2–5 cm from the thrombosis. The portal vein pressure was measured 3 times, and the mean value was taken based on the three measurements. The preoperative PPG was equal to the portal vein pressure minus the free hepatic venous pressure. The postoperative PPG was equal to the portal vein pressure minus the pressure of the inferior vena cava at the upper end of the stent.

### Data collection and follow-up

For all the patients, the following data were collected: 1. baseline data: case number, age, sex, etiology of PVT, routine blood tests, blood coagulation, blood glucose, serum creatinine, Child‒Pugh score, abdominal B-mode ultrasound, and enhanced CT; 2. intraoperative data: operation method, PPG before and after stent implantation and operation-related complications; and 3. follow-up data: blood biochemistry, liver and kidney function, main symptoms, stent patency, and patient survival. The patients were followed up regularly at 1, 3, and 6 months after TIPS and then every 6 months thereafter or whenever the patients had clinical recurrence of portal hypertension. The patients were followed up until death. The last follow-up time was September 30, 2022.

### Statistical analyses

Quantitative variables were described as the median (range), and qualitative variables were described as absolute and relative frequencies. Nonparametric testing was adopted for the median, Chi-square tests or Fisher’s exact test was used to compare frequencies and proportions, and univariate and multivariate Cox regression analyses were used to analyze the prognostic factors of survival after TIPS. The nonlinear relationships between age, Child‒Pugh score, PPG and survival were analyzed using regression curve estimation. To rule out the effect of death on rebleeding and hepatic encephalopathy, we also performed a competing risk CIF analysis for the incidence of rebleeding and hepatic encephalopathy. The Kaplan‒Meier method was used to draw the survival curve, and the log-rank test was used to compare the survival curves. Data analysis was performed using SPSS (IBM SPSS, Version 26, SPSS, Chicago, IL, USA) and Stata MP16. All tests were two sided, and a *p* value < 0.05 was considered as statistically significant.

## Results

### Patient characteristics

**Fig. 1:**
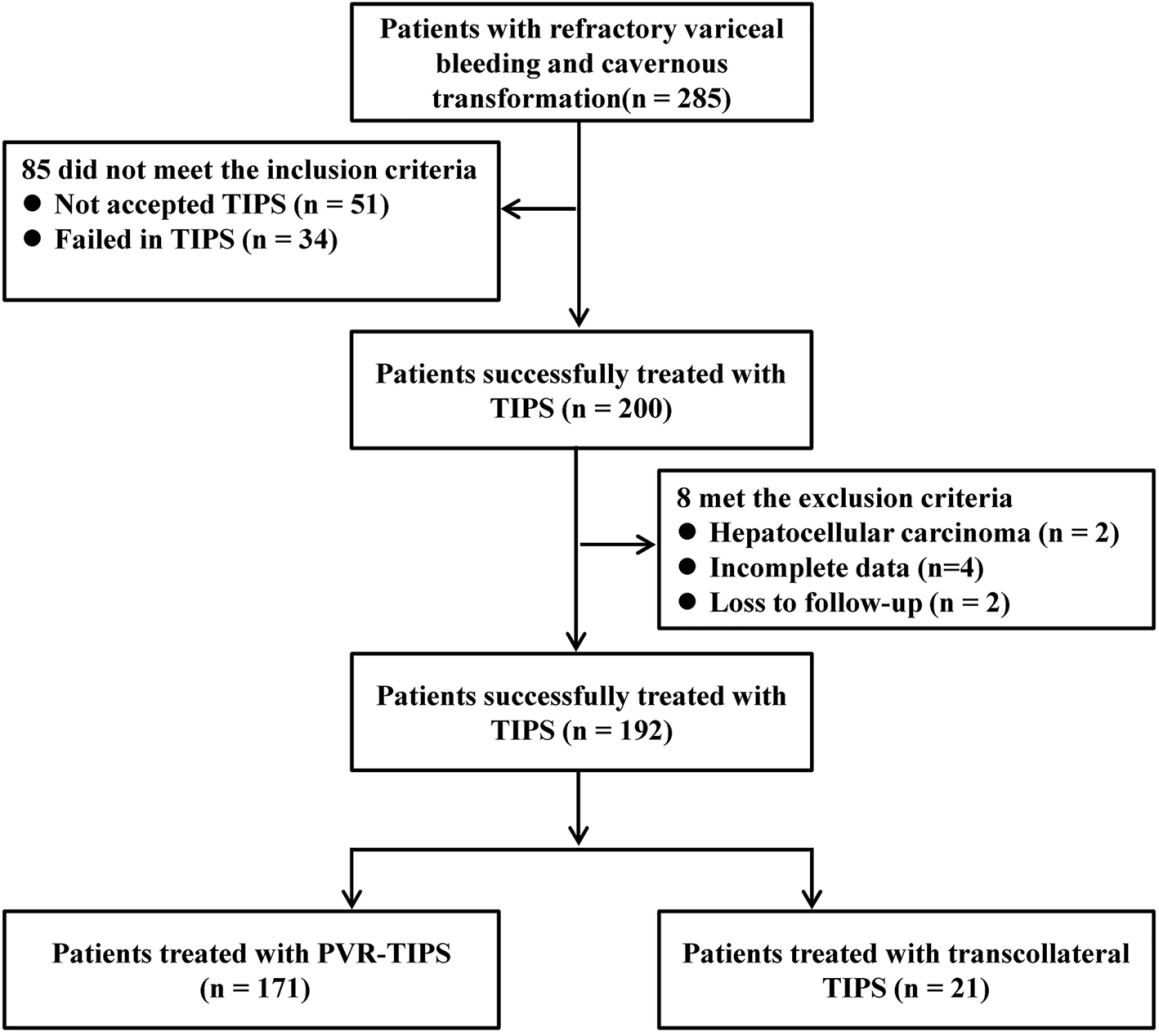
Flowchart of patient selection

From January 2015 to March 2022, there were 285 consecutive patients with refractory variceal bleeding and CTPV. Eighty-five patients did not meet the inclusion criteria. Among these, 51 patients were not treated with TIPS and 34 patients failed TIPS. Of the 51 patients without TIPS, 44 patients refused TIPS, and 7 patients were evaluated as unsuitable for TIPS. The main reason for unsuitability was the absence of available vessels for shunt creation. For 34 patients who failed TIPS, the reasons for TIPS failure were as follows: (1) there was failure in opening the main portal vein (*n* = 31) and (2) although the portal vein was opened, the patients underwent splenectomy and developed diffuse mesenteric thrombosis with sparse blood flow, making it difficult to maintain stent patency. The TIPS operation was abandoned (*n* = 2); (3) although the portal vein was opened, we could not open the splenic vein, which still had a diffuse thrombosis. Considering that even TIPS was performed, the patient still had left portal hypertension caused by splenic vein thrombosis, which could not solve the repeated variceal bleeding. The TIPS operation was abandoned (*n* = 1). Eight patients were excluded. Among them, 2 had hepatocellular carcinoma, 4 had incomplete data, and 2 were lost to follow-up. Finally, 192 patients with refractory variceal bleeding and cavernous transformation were included in this study (Fig. 1). The demographic and clinical characteristics of the 192 patients are summarized in Supplementary Table 1. One hundred and forty-six TIPS creations were performed with a “non-conventional” combined approach (transhepatic/transjugular or transsplenic/transjugular). Transhepatic approach was performed in 110 patients, and transsplenic approach was performed in 36 patients. Of the eligible patients, 171 patients (89.1%) received PVR–TIPS, and 21 patients (10.9%) received transcollateral TIPS. The patients in the transcollateral TIPS group had more noncirrhotic PVT (52.4 vs. 19.9.0, *p* = 0.002), more extensive thrombosis (38.1 vs. 15.2%, *p* = 0.026), and fewer splenectomies (14.3 vs. 40.9%, *p* = 0.018) (Table 1).

**Table 1:** Comparison between groups based on transcollateral TIPS and PVR–TIPS

Parameter median (range) or absolute (percentage)	Transcollateral TIPS (*n* = 21)	PVR–TIPS (*n* = 171)	*p* value
Age (years)	44 (10–70)	52 (14–77)	0.072
Sex			
Male	13 (61.9%)	91 (53.2%)	0.495
Female	8 (38.1%)	80 (46.8%)	
Etiology			
Liver cirrhosis	10 (47.6%)	137 (80.1%)	0.002*
Non-cirrhosis	11 (52.4%)	34 (19.9%)	
Child‒Pugh scores	7 (5–9)	7 (5–13)	0.847
Splenectomy	3 (14.3%)	70 (40.9%)	0.018*
Extent of thrombosis			
MPV	8^a^^,b^ (38.1%)	59^a^^,b^ (34.5%)	0.026*
MPV + SMV^a^	4^b^ (19.0%)	79^b^ (46.2%)	
MPV + SV	1^a^^,b^ (4.8%)	7^a^^,b^ (4.1%)	
MPV + SMV + SV	8^a^ (38.1%)	26^a^ (15.2%)	
Preoperative PPG (mmHg)	25.7 (12.5–37.9)	24.0 (8.8–40)	0.960
Postoperative PPG (mmHg)	7.4 (2.2–12.5)	8.1 (0.7–19.1)	0.718
Rebleeding from any source	3 (14.3%)	22 (12.9%)	0.741
Variceal bleeding	2 (9.5%)	20 (11.7%)	1.0
Mortality	4 (19.0%)	63 (36.8%)	0.146
Cause of death			
GI bleeding	2 (9.5%)	13 (7.6%)	
Liver failure	1 (4.8%)	12 (7.0%)	
Multiorgan failure	1 (4.8%)	5 (2.9%)	
Encephalopathy	0	13 (14.3%)	
Carcinoma	0	6 (3.5%)	
Infection	0	6 (3.5%)	
Unrelated with liver disease	0	3 (1.8%)	
Unknown	0	5 (2.9%)	

Bold values indicate a significant difference

*TIPS* transjugular intrahepatic portosystemic shunt, *PVR* portal vein recanalization, *MPV* main portal vein, *SMV* superior mesenteric vein, *SV* splenic vein, *PPG* portal pressure gradient, *GI* gastrointestinal

*p < 0.05

^a,b^Indicates the results of multiple comparisons. For those with the same letter, there is no difference between the groups

**Fig. 2:**
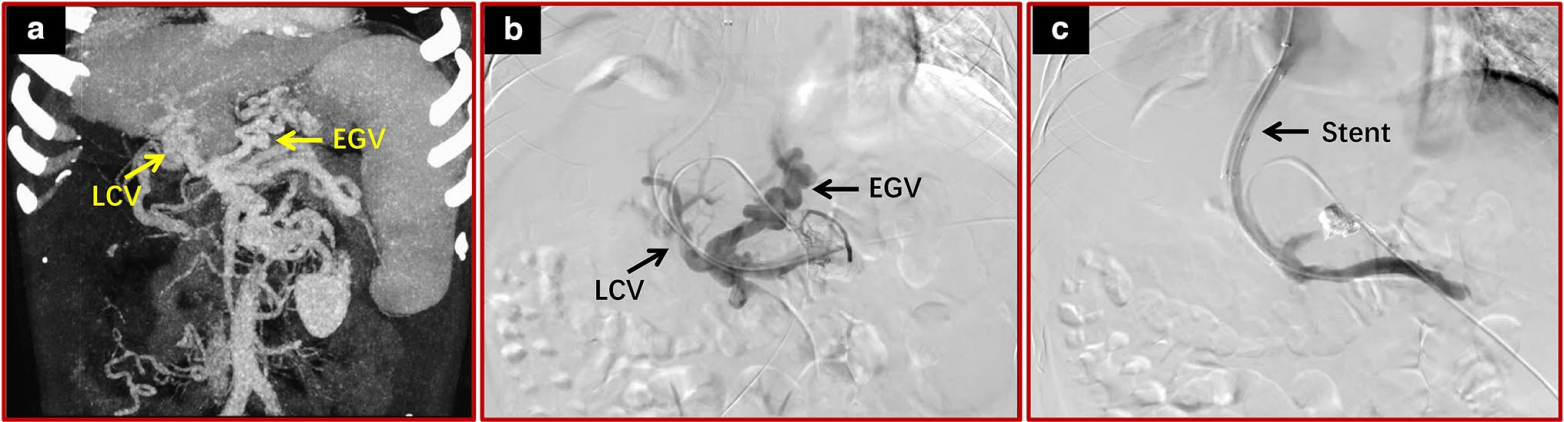
Using a collateral vessel that communicated with the variceal veins to establish a portosystemic shunt in a 50-year-old man with cirrhosis and cavernous transformation. **a** Spiral enhanced CT with multiplanar reconstruction showed cavernous transformation of the portal vein. The collateral vessels directly communicated with the variceal veins. **b** Direct portography confirmed that the targeted collateral vessels communicated with the variceal veins. **c** A stent was successfully placed between a large collateral vein and the right hepatic vein. Direct portography showed that the variceal veins had disappeared. *LCV* large collateral vessels, *EGV* esophageal and gastric varices

**Fig. 3:**
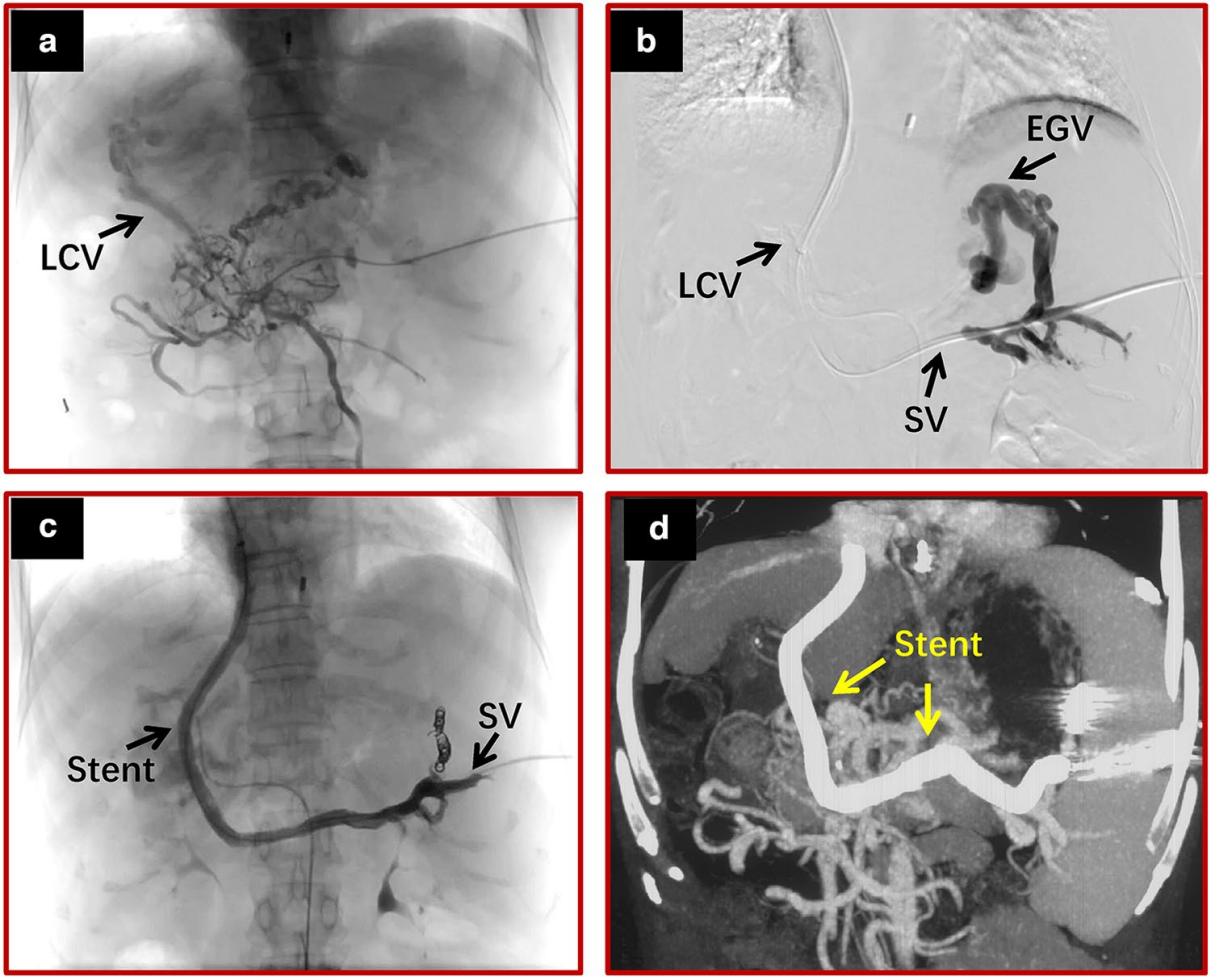
Using a collateral vessel that did not communicate with the variceal veins to establish a portosystemic shunt in a 57-year-old woman with cirrhosis and cavernous transformation. **a** Direct portography via a percutaneous transsplenic approach clearly showed that a large, cavernous collateral vessel could be used to establish a portosystemic shunt. **b** Direct splenoportography showed that the severe varicose veins did not communicate with the collateral vessel due to splenic vein thrombosis. **c** The collateral vessel was used to establish the portosystemic shunt. At the same time, the splenic vein was opened with a bare stent to make the varicose veins communicate with the collateral vessels, and the varicose veins were embolized. Direct splenoportography showed that the shunt was patent and that the varicose veins had disappeared. **d** The stents are shown on spiral enhanced CT with multiplanar reconstruction at 1 month after TIPS. *LCV* large collateral vessels, *EGV* esophageal and gastric varices, *SV* spleen vein

In the transcollateral TIPS group, there were 13 patients with varicose veins and collateral vessels directly communicating with each other. We only used a covered stent to establish a shunt between the collateral vessels and the inferior vena cava (Fig. 2). There were 8 patients with varicose veins and collateral vessels that did not directly communicate with each other. In these patients, the establishment of a collateral-systemic shunt with covered stents, as well as opening the splenic vein blocked by thrombus between the collateral branch and varicose veins with naked stents, was performed (Fig. 3). In the PVR–TIPS group, the MPV and/or blocked splenic vein were opened, and a portal-systemic shunt was established in all patients.

### Transcollateral TIPS did not increase the risk of rebleeding or death

**Fig. 4:**
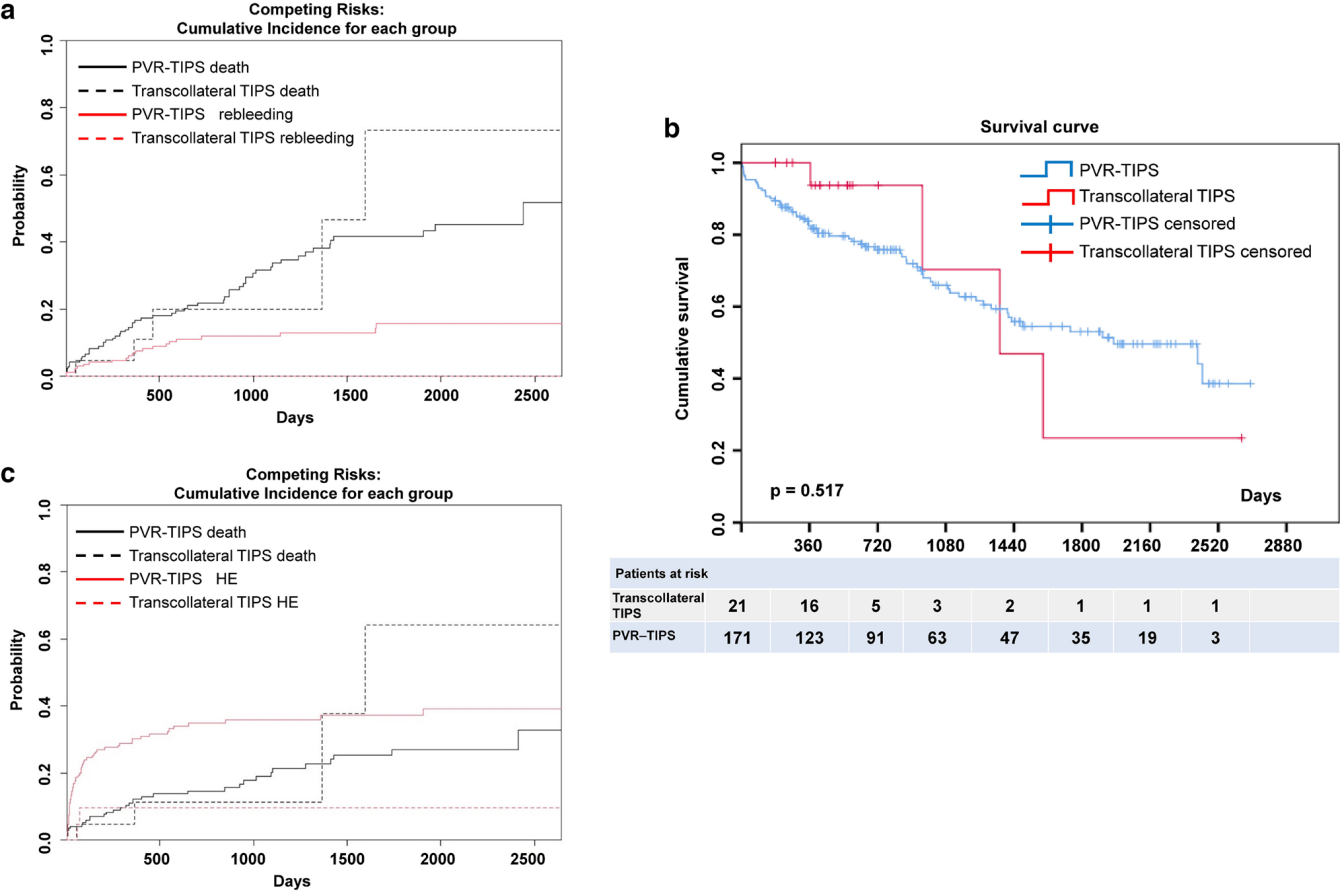
Outcome measurements were compared between the transcollateral TIPS group and the PVR–TIPS group. a Competing risk CIF analysis showed that **b** Kaplan‒Meier curves showed that the overall survival between the transcollateral TIPS group and the PVR–TIPS group was not different. **c** Competing risk CIF analysis showed that there was no significant difference in the competitive risk events between the transcollateral TIPS group and the PVR–TIPS group (*p* = 0.619). However, the incidence of encephalopathy in the transcollateral TIPS group was lower than that in the PVR–TIPS group after controlling for competitive risk events (*p* = 0.033). *PVR–TIPS* portal vein recanalization–transjugular intrahepatic portosystemic shunt, *HE* hepatic encephalopathy

There was no significant difference between PVR–TIPS and transcollateral TIPS in preoperative (25.7 vs. 24.0, *p* = 0.960), postoperative PPG (7.4 vs. 8.1, *p* = 0.960), all-cause rebleeding (14.3 vs. 12.9%, *p* = 0.741), and variceal rebleeding (9.5 vs. 11.7%, *p* = 1.0) (Table 1). Considering the competing risks of death and rebleeding, we further performed cumulative incidence for competing risk analysis. As shown in Fig. 4A, there was no significant difference in competitive risk events between the transcollateral TIPS group and the PVR–TIPS group (*p* = 0.854), and there was no significant difference in the rebleeding rate between the two groups after controlling for competitive risk events (*p* = 0.147).

Overall, 67 patients (34.9%) died during the follow-up. There were 4 deaths (19.0%) in the transcollateral TIPS group and 63 deaths (36.8%) in the PVR–TIPS group. The overall mortality rate was not significantly different between the transcollateral TIPS group and the PVR–TIPS group (19.0 vs. 36.8%, *p* = 0.106) (Table 1). Furthermore, the Kaplan‒Meier method was used to draw the survival curve, and the log-rank test was used to compare whether these survival curves were different. As shown in Fig. 4B, there was no difference in survival between the two groups (log-rank test, *p* = 0.517). Cox regression analysis was used to analyze the categorical variables associated with survival. Etiology was an independent factor associated with survival. Curve estimation was used to analyze the continuous variables related to survival. The results showed that age and Child‒Pugh score were closely related to survival. The style of TIPS had nothing to do with survival (Table 2).

**Table 2:** Analysis of survival-related factors

Variable	Univariate and multivariate cox regression analysis					
	Univariate analysis			Multivariate analysis		
	HR	95% CI	*p* value	HR	95% CI	*p* value
Sex (male)	0.849	0.524–1.376	0.506	0.934	0.573–1.522	0.783
Etiology (liver cirrhosis)	2.573	1.176–5.632	0.018*	2.629	1.162–5.946	0.020*
Splenectomy	0.847	0.518–1.384	0.507	0.759	0.461–1.249	0.278
Extent of thrombosis (MPV)	1.148	0.688–1.914	0.597	1.063	0.633–1.786	0.817
Style of TIPS (PVR–TIPS)	0.716	0.259–1.977	0.519	0.950	0.331–2.729	0.924

	Curve estimation		
	Models	Adjusted *R*^2^	*p* value
Age	S	0.046	0.003*
Child‒Pugh score	Logarithmic	0.066	0.000*
Preoperative PPG (mmHg)	S	0.000	0.870
Postoperative PPG (mmHg)	S	0.003	0.452

Bold values indicate a significant difference

*MPV* main portal vein, *TIPS* transjugular intrahepatic portosystemic shunt, *PVR* portal vein recanalization, *PPG* portal pressure gradient

*p < 0.05

### Transcollateral TIPS did not increase the incidence of complications

There were no significant differences between the transcollateral TIPS group and the PVR–TIPS group in the incidence of operation-related complications. The operation-related complications included intraperitoneal bleeding, subcutaneous hematoma at the puncture site, and ectopic embolism (Supplementary table 2). One patient treated with PVR–TIPS had a splenic vein injury and was treated with splenectomy. The remaining patients with operation-related complications were treated with drugs or blood transfusions or were observed to be cured. There were no deaths from operation-related complications.

The nonoperation-related complications included TIPS stenosis, hepatic encephalopathy, liver function damage, acute episodes of chronic liver failure, fever, hepatocellular carcinoma, and death within 6 weeks after TIPS. Overt hepatic encephalopathy was significantly lower in the transcollateral TIPS group (9.5 vs. 35.1%, *p* = 0.018). Similar results were obtained using competing risk CIF analysis. As shown in Fig. 4C, there was no significant difference in competitive risk events between the transcollateral TIPS group and the PVR–TIPS group (*p* = 0.619). However, the incidence of encephalopathy in the transcollateral TIPS group was lower than that in the PVR–TIPS group after controlling for competitive risk events (*p* = 0.033). In terms of other nonoperation-related complications, there were no significant differences between the transcollateral TIPS group and the PVR–TIPS group (Supplementary table 2).

## Discussion

CTPV with complications of portal hypertension is a difficult clinical problem. For the treatment of thrombus itself, the anticoagulant effect is poor; for the treatment of portal hypertension complications caused by thrombus, traditional methods such as drugs and/or endoscopy are ineffective. Liver transplantation is difficult to perform [15, 21, 22]. PVR–TIPS offers new hope for the treatment of these patients. However, PVR–TIPS is a very difficult operation even for experienced surgeons, and the success rate is unlikely to reach 100%. The use of the collateral vein to establish a shunt is an alternative method when recanalization of the portal vein is not feasible, but its effectiveness is unclear. In this study, patients with refractory esophageal and/or gastric variceal bleeding caused by portal cavernous formation were selected from the prospective database of consecutive patients treated with TIPS in Xijing Hospital from January 2015 to March 2022. According to the method of TIPS, the patients were divided into the transcollateral TIPS group and the PVR–TIPS group. The efficacy and safety of these two methods of TIPS for CTPV with refractory variceal bleeding were analyzed. The results showed that transcollateral TIPS did not increase the risk of rebleeding, death, or operation-related complications. Furthermore, overt hepatic encephalopathy was significantly lower in the transcollateral TIPS group than in the PVR–TIPS group. These results concluded that transcollateral TIPS is a safe and effective treatment for portal cavernous formation with refractory variceal bleeding. All patients in the transcollateral TIPS group presented with recurrent variceal bleeding after endoscopic and medical treatment and received TIPS as salvage therapy. They were all treated with transcollateral TIPS when the portal vein could not be opened. To our knowledge, this is the first well-documented study of transcollateral TIPS.

This study confirmed that transcollateral TIPS was effective in the treatment of CTPV with recurrent variceal bleeding. We successfully treated CTPV with variceal bleeding using transcollateral TIPS [15]. Yamagami et al*.* successfully treated a 65-year-old woman with recurrent variceal bleeding due to CTPV by using transcollateral TIPS, and no subsequent portal hypertensive bleeding occurred after transcollateral TIPS [16]. Brountzos et al. treated a 72-year-old patient with refractory portal hypertensive ascites due to CTPV using transcollateral TIPS. The ascites disappeared after the operation, the stent was unobstructed, and no recurrence of ascites occurred during the follow-up period of 16 months [17]. However, Alexandra Wils reported 4 patients who were treated with transcollateral TIPS, 2 of whom had good results, and 2 patients died within a short period after TIPS. One death occurred shortly after TIPS because of rebleeding of gastric varices. Another death was due to acute respiratory distress syndrome complicated by cardiac arrhythmias and bradycardia. The authors concluded that bleeding can be prevented for variceal veins communicating with collateral branches but not for variceal veins that do not communicate with collateral branches [23]. Another researcher used the method of transcollateral TIPS to treat a patient with recurrent variceal bleeding and CTPV. Variceal bleeding still occurred repeatedly after TIPS. The authors concluded that the pressure reduction of the portal system was not sufficient in patients with transcollateral TIPS, so rebleeding still occurred after the operation [14]. The reasons for the different results in those previous patients might be as follows: 1. the different conditions of the collateral vessels used for the shunt; and 2. the various relationships between the variceal veins and collateral vessels. If the collateral vessels had a larger diameter and were directly communicating with variceal veins, the PPG decreased more, and the risk of rebleeding was lower. Otherwise, it was the opposite. Therefore, we defined the collateral vessels used to establish portosystemic shunts for the first time, with a diameter of more than 6 mm and a straight vessel of more than 2 cm. The main reason was that the diameter of the TIPS stent used in the operation was 8 mm, and a 6–8 mm balloon was used to expand the stent after implantation. If the collateral vessels were less than 6 mm, the stent might not be sufficiently dilated, and the PPG might not be sufficiently decreased. In addition, there was a 2 cm bare area at the lower end of the TIPS stent, and a 2 cm straight blood vessel was required to conform to the stent bare area and that the fit was adequate. Furthermore, the vessels (usually the splenic vein) between the target collateral and the variceal veins should be opened if the collateral vessel does not directly communicate with the variceal veins. For these patients, it is not enough to establish a shunt using a collateral vessel. The blood from the varicose veins and their nourishing vessels does not directly flow into the shunt, and there is still local portal hypertension. Therefore, it is necessary to open the vessels between the varicose veins and the collateral vessels while establishing the shunt using the collateral vessels (Fig. 3). Because we defined the conditions for collateral vessels and opened the vessels between the collateral and varicose veins, the effect of collateral TIPS in preventing rebleeding was not different from that of PVR–TIPS.

This study also verified that transcollateral TIPS was safe. The collateral vessels of the cavernous transformation, unlike the portal vein, are not surrounded by the Glison sheath. They are often located outside the liver. Therefore, when using collateral vessels to perform TIPS, the operator is mostly worried about liver and collateral vessel injury leading to massive abdominal bleeding. Indeed, transcollateral TIPS is more difficult than conventional TIPS. One key point is that the number of punctures must be minimized. Second, when the external sheath of the RUPS100 is delivered to the collateral vessels, the supporting force of the guide wire should be sufficient. If the hard guide wire is not deep enough, the external sheath of the RUPS100 cannot be sent to the collateral vessels. At this time, other covered stents can be used instead of GOREVIATORR. Therefore, although the transcollateral TIPS technique is harder, for experienced experts, transcollateral TIPS does not increase operation-related complications compared with PVR–TIPS. In our study, the number of operation-related complications and overall survival of transcollateral TIPS were comparable to those of PVR–TIPS. Furthermore, our study found that the incidence of hepatic encephalopathy in the transcollateral TIPS group was significantly lower than that in the PVR–TIPS group. This may be because the blood of the portal vein was often completely shunted in the PVR–TIPS group but was only partly shunted in the transcollateral TIPS group. There may be other collateral veins to supply blood to the liver in transcollateral TIPS. In summary, the results of this study preliminarily confirmed that transcollateral TIPS is safe and effective in the treatment of CTPV with recurrent variceal bleeding.

For noncirrhotic portal vein thrombosis, if only extrahepatic portal vein obstruction but the intrahepatic portal vein is patent, only portal vein recanalization should be performed instead of TIPS. However, for patients with extrahepatic and intrahepatic portal vein thrombosis, simple portal vein recanalization is usually ineffective. In this study, 45 patients with noncirrhotic portal thrombosis enrolled were all unsuitable for portal vein recanalization because of thrombi in the secondary branches of the intrahepatic portal vein. Therefore, they were treated with TIPS.

This study had the following limitations. First, this was a retrospective study. Second, in all patients, transcollateral TIPS was chosen when the portal vein could not be opened rather than both PVR–TIPS and transcollateral TIPS. Therefore, this study has some bias. That is, the degree of PVT in patients with collateral TIPS is more severe than that in patients with PVR–TIPS. Third, the sample size of transcollateral TIPS was small.

In conclusion, our study suggests that transcollateral TIPS is a safe and effective alternative for patients with CTPV and recurrent variceal bleeding who have difficulty implementing PVR–TIPS. At the same time, in this study, we preliminarily proposed the technical standard of transcollateral TIPS, namely, the diameter of the collateral is greater than 6 mm, and the straight length of the collateral is more than 2 cm. Moreover, the vessels that give off the variceal veins should be opened to directly communicate with the collateral vessels.

## Supplementary Information

Below is the link to the electronic supplementary material.

Supplementary file1 (DOCX 58 KB)

Supplementary file2 (JPG 1371 KB)

Supplementary file3 (JPG 891 KB)

Supplementary file4 (JPG 1657 KB)

Supplementary file5 (PPTX 114551 KB)

## Data Availability

The raw data supporting the conclusions of this article will be made available by the authors.
