# Supplementary material for: Transjugular intrahepatic collateral-systemic shunt is effective for cavernous transformation of the portal vein with variceal bleeding
Source: Hepatol Int. 2023 Apr 25;17(4):979–88. doi: 10.1007/s12072-023-10522-z (PMC10386942; doi:10.1007/s12072-023-10522-z)
Supplement: Supplementary file 1 — Supplementary file1 (DOCX 58 KB) [file 12072_2023_10522_MOESM1_ESM.docx]

**Supplementary figure legends**

**Supplementary figure 1. Pictorial depiction of TIPS eligibility in patients with CTPV.** (A) Spiral enhanced CT with multiplanar reconstruction (MPR) showed occlusive PVT with patency of the splenic vein and mesenteric vein. (B) Complete portal and mesenteric vein thrombosis with distal patency of the splenic vein. (C) Complete portal vein thrombosis and splenectomy with distal patency of the mesenteric vein. (D) CTPV and diffuse thrombosis of the splenic vein and mesenteric vein. TIPS eligibility in patients with CTPV included in (A), (B), and (C). The image of (D) was ineligible for TIPS. Abbreviations: TIPS, transjugular intrahepatic portosystemic shunt; CTPV, cavernous transformation of the portal vein; MPV, main portal vein; SV, splenic vein; SMV: superior mesenteric vein.

**Supplementary figure 2. Pictorial depiction of the collateral vessels used to establish portosystemic shunts.** (A) Spiral enhanced CT with multiplanar reconstruction (MPR) showed occlusive PVT with cavernous transformation. The main portal vein was completely occluded with thrombosis. There were some collateral vessels around the portal veins. (B) One of them had a diameter of more than 6 mm and a straight vessel segment of more than 2 cm. (C) Indirect portal vein angiography confirmed occlusive PVT with cavernous transformation. (D) MPR at 6 months after transcollateral TIPS showed that the stent was fluent. Abbreviations: LCV, large collateral vessels; OPVT, occlusive portal vein thrombosis; SVS, straight vessel segment.

**Supplementary** **figure 3. The location of the portal vein pressure measurement in patients with CTPV.** (A) For patients with transcollateral TIPS and whose collateral vessel directly communicated with variceal veins, the portal pressure measurement site was located 2-5cm distal to the collateral vessel puncture site. The portal pressure measurement sites were located in superior mesenteric vein (B), or splenic vein (C) 2-5cm distal to the thrombus for patients with occlusive PVT with patency of the splenic vein and mesenteric vein, splenic vein 2-5cm distal to the thrombus for patients with CTPV and diffuse thrombosis of mesenteric vein (D), or superior mesenteric vein 2-5cm distal to the thrombus for patients with CTPV and splenectomy (E).

**Supplementary tables**

**Supplementary table 1. Demographic and clinical characteristics of the 192 patients**

| Parameter  Median (range) or absolute (percentage) |  | Value |
| --- | --- | --- |
| Median Age (years) |  | 51 (10-77) |
| Sex |  |  |
| Male |  | 104 (54.2%) |
| Female |  | 88 (45.8%) |
| Etiology |  |  |
| Liver cirrhosis |  | 147 (76.6%) |
| Noncirrhosis |  | 45 (23.4%) |
| Liver function score |  |  |
| Child‒Pugh |  | 7 (5-13) |
| Child‒Pugh class A/B/C |  | 71/105/16 |
| Splenectomy |  |  |
| Yes |  | 73 (38.0%) |
| No |  | 119 (62.0%) |
| Extent of thrombosis |  |  |
| MPV |  | 67 (34.9%) |
| MPV+SMV |  | 83 (43.2%) |
| MPV+SV |  | 8 (4.2%) |
| MPV+SMV+ SV |  | 34 (17.7%) |
| Style of TIPS |  |  |
| PVR–TIPS |  | 171 (89.1%) |
| Transcollateral TIPS |  | 21 (10.9%) |
| Preoperative PPG (mmHg) (IQR) |  | 24.0 (20.0-28.7) |
| Postoperative PPG (mmHg) (IQR) |  | 8.1 (0.7-19.1) |

MPV, main portal vein; SMV: superior mesenteric vein; SV, splenic vein; TIPS, transjugular intrahepatic portosystemic shunt; PVR, portal vein recanalization; PPG, portal pressure gradient; IQR, interquartile range.

**Supplementary table 2. Complications of the transcollateral TIPS and PVR–TIPS**

| **Complications** | **Transcollateral TIPS**  **(n = 21)** | **PVR–TIPS**  **(n = 171)** | **P Value** |
| --- | --- | --- | --- |
| **Operation-related complications** |  |  |  |
| Intraperitoneal bleeding | 1 (4.8%) | 7 (4.1%) | 1.0 |
| Subcutaneous hematoma | 0 (0%) | 3 (1.8%) | 1.0 |
| Ectopic embolism | 1 (4.8%) | 6 (3.5%) | 0.562 |
| **Nonoperation-related complications** |  |  |  |
| TIPS stenosis, n (%) | 4 (19.0%) | 40 (23.4%) | 0.788 |
| Asymptomatic TIPS stenosis | 2 (9.5%) | 18 (15.4%) | 0.813 |
| Overt hepatic encephalopathy, n (%) | 2 (9.5%) | 60 (35.1%) | **0.024** |
| More than one episode | 1 (4.8%) | 38 (22.2%) | 0.082 |
| Grade 3-4 | 2 (9.5%) | 16 (9.4%) | 1.0 |
| Liver function damage |  |  |  |
| 1 month after TIPS | 1/21 (4.8%) | 12/164 (7.3%) | 1.0 |
| 3 months after TIPS | 1/20 (4.8%) | 8/158 (5.1%) | 1.0 |
| 6 months after TIPS | 0/20 (0%) | 3/153 (2.0%) | 1.0 |
| Acute episode in chronic liver failure | 2 (9.5%) | 17 (9.9%) | 1.0 |
| Fever | 2 (9.5%) | 14 (8.2%) | 0.689 |
| Hepatocellular carcinoma | 0 (0%) | 17 (9.9%) | 0.225 |
| Death within 6 weeks after TIPS | 0 (0%) | 8 (4.7%) | 0.602 |

TIPS, transjugular intrahepatic portosystemic shunt; PVR, portal vein recanalization.
