# Supplementary figures and images for: Transjugular intrahepatic collateral-systemic shunt is effective for cavernous transformation of the portal vein with variceal bleeding
Source: Hepatol Int. 2023 Apr 25;17(4):979–88. doi: 10.1007/s12072-023-10522-z (PMC10386942; doi:10.1007/s12072-023-10522-z)

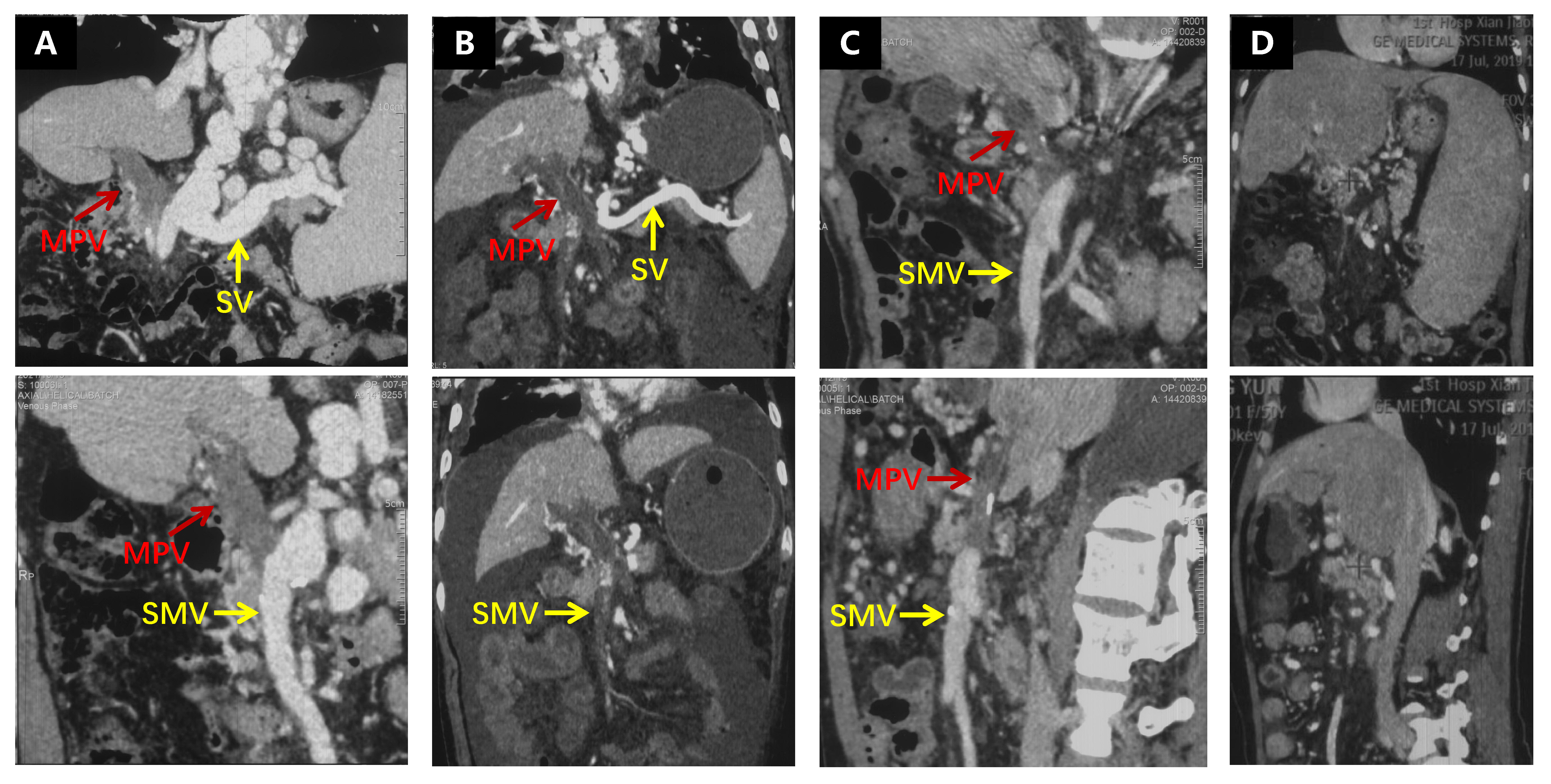

Supplement: Supplementary file 2 — Supplementary file2 (JPG 1371 KB) [file 12072_2023_10522_MOESM2_ESM.jpg]

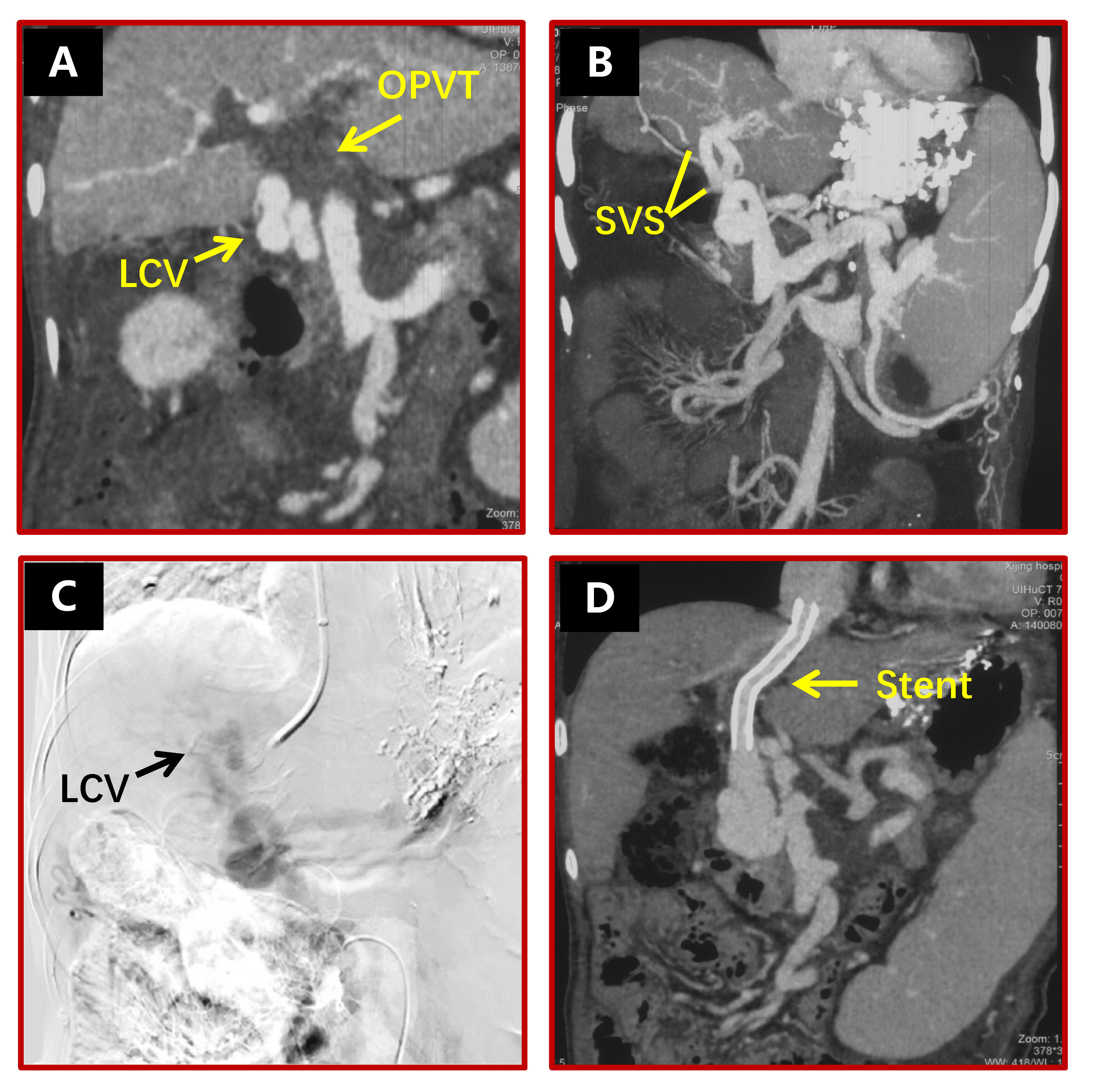

Supplement: Supplementary file 3 — Supplementary file3 (JPG 891 KB) [file 12072_2023_10522_MOESM3_ESM.jpg]

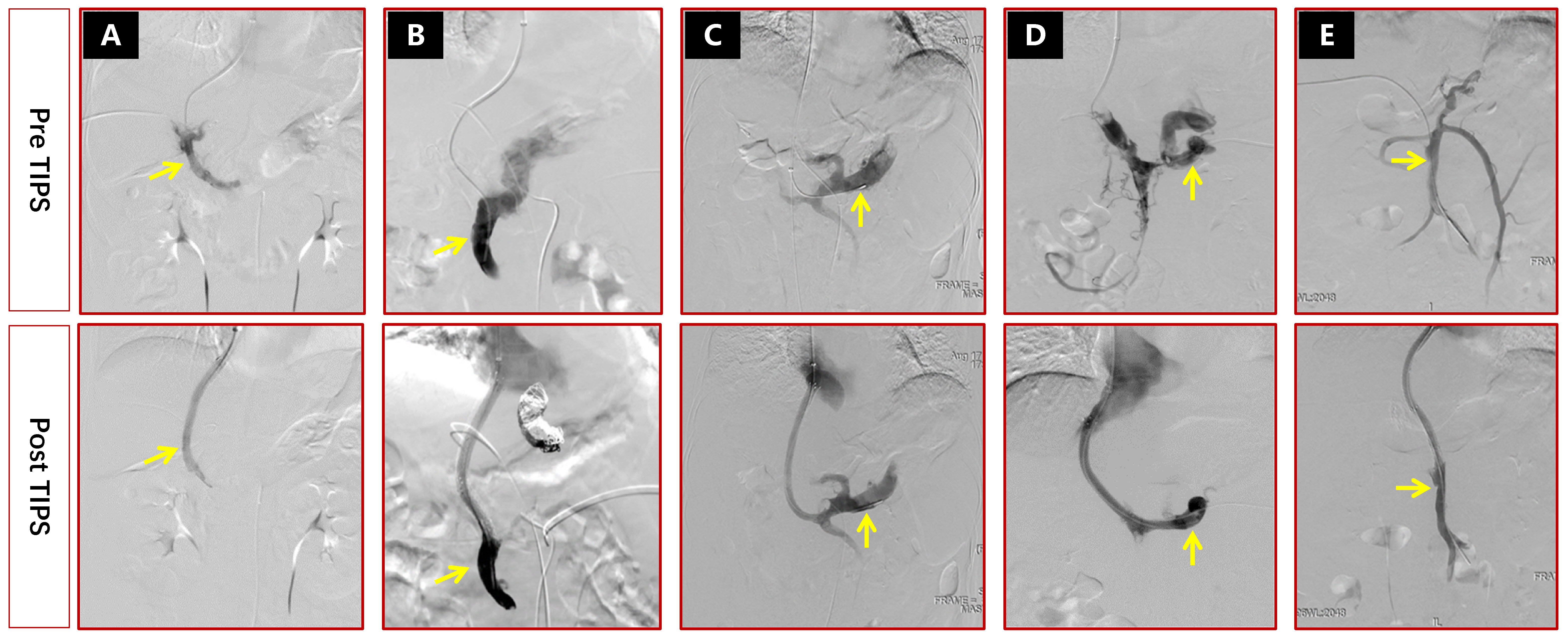

Supplement: Supplementary file 4 — Supplementary file4 (JPG 1657 KB) [file 12072_2023_10522_MOESM4_ESM.jpg]
